# The Development of Novel Compounds Against Malaria: Quinolines, Triazolpyridines, Pyrazolopyridines and Pyrazolopyrimidines

**DOI:** 10.3390/molecules24224095

**Published:** 2019-11-13

**Authors:** Luiz C. S. Pinheiro, Lívia M. Feitosa, Marilia O. Gandi, Flávia F. Silveira, Nubia Boechat

**Affiliations:** 1 Departamento de Síntese de Fármacos, Instituto de Tecnologia em Fármacos, Farmanguinhos-FIOCRUZ, Fundação Oswaldo Cruz, Rua Sizenando Nabuco 100, Manguinhos, Rio de Janeiro 21041-250, Brazillivia.feitosarj@gmail.com (L.M.F.); mariliaolivagandi@gmail.com (M.O.G.); flaviafernandes_jo23@hotmail.com (F.F.S.); 2 Programa de Pós-Graduação em Farmacologia e Química Medicinal, PPGFQM, Instituto de Ciências Biomédicas, Universidade Federal do Rio de Janeiro, Rio de Janeiro 21041-250, Brazil; 3 Programa de Pós-Graduação em Química, PGQu Instituto de Química, Universidade Federal do Rio de Janeiro, Rio de Janeiro 21041-250, Brazil

**Keywords:** malaria, *P. falciparum*, *Pf*DHODH, quinoline, triazolopyrimidine, pyrazolopyridine, pyrazolopyrimidine, chloroquine, primaquine, mefloquine

## Abstract

Based on medicinal chemistry tools, new compounds for malaria treatment were designed. The scaffolds of the drugs used to treat malaria, such as chloroquine, primaquine, amodiaquine, mefloquine and sulfadoxine, were used as inspiration. We demonstrated the importance of quinoline and non-quinoline derivatives in vitro with activity against the W2 chloroquine-resistant (CQR) *Plasmodium falciparum* clone strain and in vivo against *Plasmodium berghei*-infected mouse model. Among the quinoline derivatives, new hybrids between chloroquine and sulfadoxine were designed, which gave rise to an important prototype that was more active than both chloroquine and sulfadoxine. Hybrids between chloroquine–atorvastatin and primaquine–atorvastatin were also synthesized and shown to be more potent than the parent drugs alone. Additionally, among the quinoline derivatives, new mefloquine derivatives were synthesized. Among the non-quinoline derivatives, we obtained excellent results with the triazolopyrimidine nucleus, which gave us prototype **I** that inspired the synthesis of new heterocycles. The pyrazolopyrimidine derivatives stood out as non-quinoline derivatives that are potent inhibitors of the *P. falciparum* dihydroorotate dehydrogenase (*Pf*DHODH) enzyme. We also examined the pyrazolopyridine and pyrazolopyrimidine nuclei.

## 1. Introduction

Malaria is one of the world’s most serious public health problems. According to the latest World Health Organization (WHO) World Malaria Report, no significant gains were achieved in reducing malaria cases in the period from 2015 to 2017. The estimated number of malaria deaths in 2017, at 435,000, remained broadly unchanged relative to the previous year, which was 445,000 deaths (WHO, 2019) [1].

Due to the high parasitic resistance exhibited by *Plasmodium falciparum* to most drugs available, monotherapy is no longer used to treat malaria.

To prevent recurrence and delay the development of parasite resistance, the WHO recommends the use of artemisinin-combined therapies (ACTs), which are based on the simultaneous use of drugs with different modes of action [2]. Artemisinin derivatives have very short in vivo half-lives, but they are fast acting against the intraerythrocytic asexual blood-stage malaria parasites. Therefore, artemisinin derivatives are coadministered with drugs with longer half-lives [2]. There is an urgent need for novel antimalarials with better safety profiles than current medicines due to the resistance against antimalarial drugs and for the prevention of transmission and relapse of the disease [3,4,5,6].

In the recent literature, a number of new antimalarial compounds in different stages of preclinical and clinical development have been described [7,8,9,10]. Notably, quinoline derivatives are still the predominant class of antimalarial drugs. [11,12,13].

The exchange of the quinoline ring with another heterocyclic ring is an important strategy in drug design and the chemical modification of available drugs to develop novel, biologically active compounds. Many heterocyclic compounds have been developed in an attempt to find new drugs to treat malaria [14].

**Figure 1:**
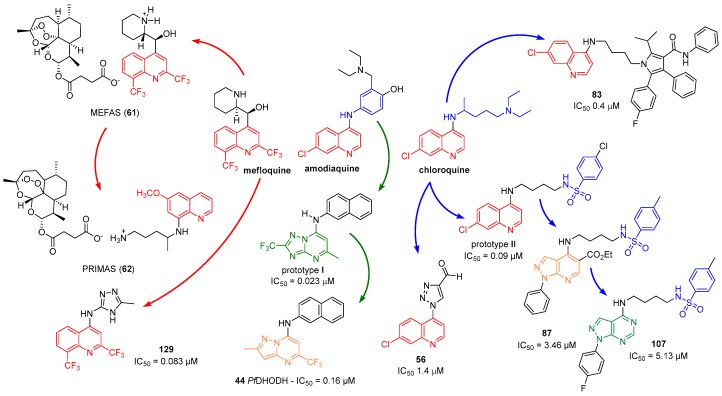
Rational approach to the design quinoline and non-quinoline compounds.

Recent studies by Boechat and coworkers present the design and synthesis of a broad class of quinoline and non-quinoline compounds with anti-*P falciparum*. These compounds had their structures modified using some medicinal chemistry tools, such as molecular hybridization. The rational sequence of modifications made from the parent drugs and/or prototypes to obtain the novel hybrid compounds is summarized in Figure 1.

**Figure 2:**
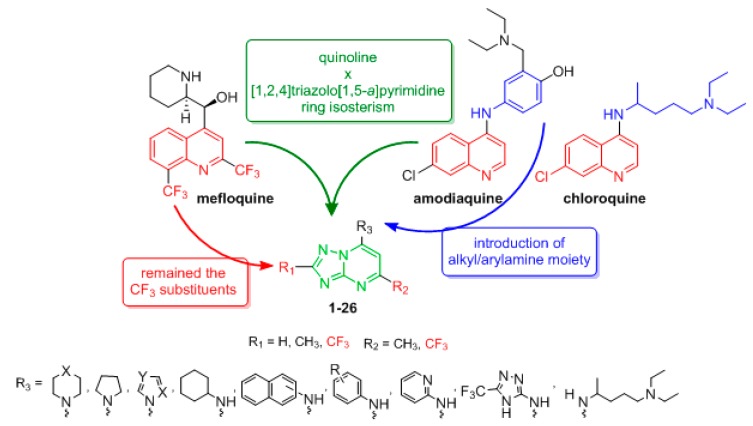
Rational approach to the design of [1,2,4]triazolo[1,5-*a*]pyrimidine derivatives **1**–**26**.

Therefore, twenty-six new derivatives of the [1,2,4]triazolo[1,5-*a*]pyrimidine system (**1**–**26**), with different substituents at the 2, 5 and 7 positions, were designed using standard medicinal chemistry and modeling principles, such as isosteric replacement, based on ring isosterism with the antimalarial drugs mefloquine, chloroquine and amodiaquine (Figure 2) [15]. Additionally, the CF_3_ group present in mefloquine was added at the 2 position of the triazolopyrimidine ring, and aromatic and aliphatic amine moieties were incorporated at the 7 position, taking into consideration the amodiaquine scaffold. The trifluoromethyl moiety is one of the most widespread fluorine-containing functional groups in bioactive molecules. Due to its highly electronegative feature, it can exert significant electronic influences on neighboring groups. Another advantage is the improvement in lipophilicity, making this moiety useful for targeting molecules to enzymatic active sites [16,17,18].

**Figure 3:**
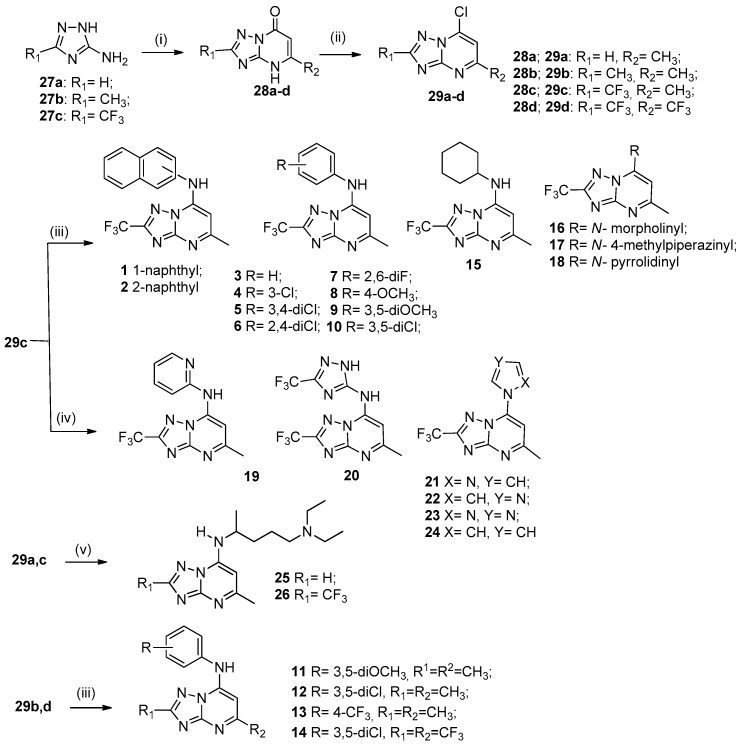
Synthesis of [1,2,4]triazolo[1,5-*a*]pyrimidines **1**–**26**. *Reagents and conditions:* (i) ethyl acetoacetate or ethyl 4,4,4-trifluoroacetoacetate, TsOH (cat.), toluene, reflux, 24 h; (ii) POCl_3_, reflux, 6 h; (iii) appropriate amine, EtOH, 25 °C, 16–18 h; (iv) appropriate amine/azol, DMF, 120 °C, 12 h; (v) appropriate amine, EtOH, 25 °C, 43 h.

The reaction of 3-amino-1,2,4-triazoles **27a**–**c** with ethyl acetoacetate or ethyl 4,4,4-trifluoroacetoacetate in toluene under reflux with catalytic *p*-toluenesulfonic acid, for 24 h produced [1,2,4]triazolo[1,5-*a*]pyrimidin-7(4*H*)-ones **28a**–**d** in 50–90% yield [19]. Compounds **28a**–**d** were treated with POCl_3_ under reflux for 6 h to obtain the respective 7-chloro[1,2,4]triazolo[1,5-*a*]pyrimidines **29a**–**d** in 58–90% yield. The nucleophilic aromatic substitution reaction of intermediates **29a**–**d** with the appropriate amines [20] afforded target compounds [1,2,4]triazolo[1,5-*a*]pyrimidine derivatives **1**–**26** in 30–90% yield (Figure 3).

**Figure 4:**
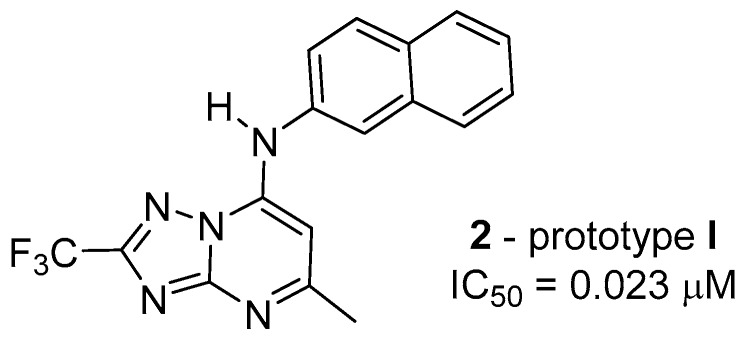
Structure of prototype **I**.

Synthesized compounds **1**–**26** were evaluated in vitro against the W2 chloroquine-resistant (CQR) *P. falciparum* clone strain, which showed IC_50_ values in the range of 0.023 to 20 μM and did not present toxicity to HepG2 cells. The trifluoromethyl group, as a substituent at the 2 position of the [1,2,4]triazolo[1,5-*a*]pyrimidine ring, contributed to an increase in anti-*P. falciparum* activity. Compounds **2** (2-naphthyl; IC_50_ = 0.023 μM), **5** (3,4-diCl; IC_50_ = 0.55 μM), **8** (4-OCH_3_; IC_50_ = 0.4 μM), and **13** (4-CF_3_; IC_50_ = 0.3 μM) were the most potent of the series. However, the 5-methyl-7-*N’*-(*N*,*N*-diethylpentane-1,4-diamine)-2-(trifluoromethyl)[1,2,4]triazolo[1,5-*a*]pyrimidine derivative that contained a trifluoromethyl group showed poor antimalarial activity. Derivative **2**, which contains a β-naphthylamine group in its structure at the 7 position, and the trifluoromethyl group at the 2 position, has an important contribution to anti-*P. falciparum* activity when compared with the other derivatives that contain aryl/alkylamine groups at the 7 position. Derivative **2** was therefore used as a prototype compound (prototype **I**) for future investigations in the search for compounds for the treatment of malaria (Figure 4).

**Figure 5:**
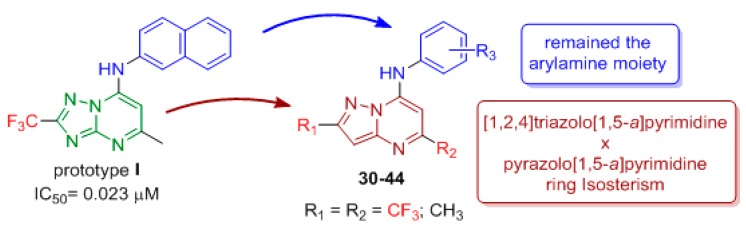
Rational approach to the design of pyrazolo[1,5-*a*]pyrimidine derivatives **30**–**44**.

The inhibition of *P. falciparum* dihydroorotate dehydrogenase (*Pf*DHODH) has been shown to be an attractive strategy to search for new substances with antiplasmodial activity [21,22]. Synthetic compounds have been identified as inhibitors of class 2 DHODH enzymes. Some [1,2,4]triazolo[1,5-*a*]pyrimidin-7-amines were discovered by Phillips and coworkers as inhibitors of *Pf*DHODH [23,24,25,26]. Following these good results, new 7-arylaminopyrazolo[1,5-*a*]pyrimidines were designed by ring isosterism from prototype **I** [27]. The [1,2,4]triazolo[1,5-*a*]pyrimidine ring was modified to a pyrazolo[1,5-*a*]pyrimidine ring and changed by molecular hybridization (Figure 5).

Different arylamines were incorporated into the structure to investigate the importance of the substituent at the 7 position of the pyrazolopyrimidine nucleus, and the β-naphthylamine moiety was also incorporated. In a previous work, we observed that a trifluoromethyl group at the 2 position of the triazolopyrimidine ring plays an important role in antiplasmodial activity. This time, CF_3_ was also added as a substituent in the 2 or 5 position of the pyrazolo[1,5-*a*]pyrimidine scaffold to investigate the influence of this group on the anti-*P. falciparum* activity.

**Figure 6:**
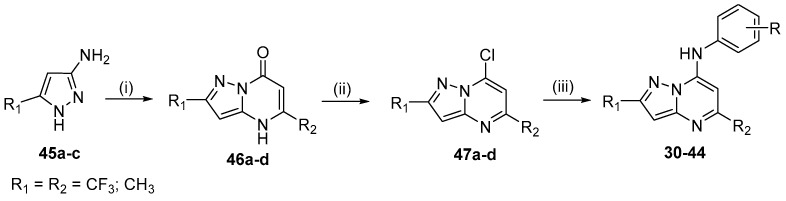
Synthesis of pyrazolo[1,5-*a*]pyrimidines **30**–**44**. *Reagents and conditions*: (i) ethyl acetoacetate or ethyl 4,4,4-trifluoroacetoacetate, TsOH (cat.), toluene, reflux, 20 h; (ii) POCl_3_, reflux, 4 h; (iii) appropriate amine, EtOH, 25 °C, 16–18 h.

Fifteen compounds, **30**–**44**, were synthesized in 44%–92% yield using the same synthetic methodology described above (Figure 6) [15].

These compounds were evaluated in vitro against the *P. falciparum* W2 CQR clone, in vivo against *P. berghei*-infected mouse model, and in vitro as *Pf*DHODH inhibitors. In addition, a molecular docking study was performed to evaluate the possible binding mode of the 7-arylaminopyrazolo[1,5-*a*]pyrimidine compounds to *Pf*DHODH.

Among the 15 pyrazolopyrimidines synthesized, **13** exhibited anti-*P. falciparum* activity, with IC_50_ values ranging from 1.2 to 92.4 μM. Compounds **33** (R_1_ = CF_3_, R_2_ = CH_3_), **38** (R_1_ = CH_3_, R_2_ = CH_3_) and **44** (R_1_ = CH_3_, R_2_ = CF_3_) with β-naphthylamine at the 7 position were the most active. Compounds **33** and **38** exhibited low toxicity and low IC_50_ values of 1.2 and 5.1 μM, respectively, and consequently, the highest SI (selectivity index) values of 467.8 and 79.6, respectively. Therefore, compounds **33** and **38** were selected for in vivo in *P. berghei*-infected mouse model. On day 5 upon treatment at 5 mg/kg, administered orally, both compounds reduced parasitemia by 50%. Compound **44** showed the best inhibition of the *Pf*DHODH enzyme with an IC_50_ = 0.16 μM, which was more potent than prototype **I** (IC_50_ = 0.70 μM), whereas **33** and **38** had IC_50_ values of 6.0 and 4.0 μM, respectively.

**Figure 7:**
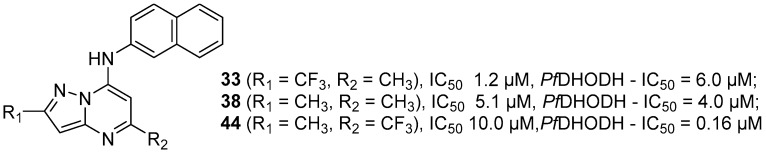
Structure of pyrazolo[1,5-*a*]pyrimidines **30**–**44**.

Compounds that had better activity against *P. falciparum* were also the most active against the *Pf*DHODH enzyme (Figure 7), which indicated that inhibition of this enzyme was one of the potential mechanisms of action of the 7-arylaminepyrazolo[1,5-*a*]pyrimidines.

The proposed bioisosteric replacement of the [1,2,4]triazolo[1,5-*a*]pyrimidine ring on prototype **I** by the pyrazolo[1,5-*a*]pyrimidine ring was shown to be a positive proposition according to the molecular docking study. These results demonstrated the potential of 7-arylpyrazolo[1,5-*a*]pyrimidine derivatives as inhibitors of *Pf*DHODH and may represent new leads for developing drugs against malaria.

Although quinoline derivatives continue to dominate the antimalarial drug, new synthetic hybrid compounds have been described in recent literature [28,29,30,31,32,33,34]; however, due to resistance to this class of drugs, searching for other analogous compounds is extremely important [35].

In an effort to obtain novel quinoline derivatives for the treatment of malaria, new quinolinyl-1*H*-1,2,3-triazoles (**48**–**58**) were designed to find new compounds with anti-*P. falciparum* activity [36].

**Figure 8:**
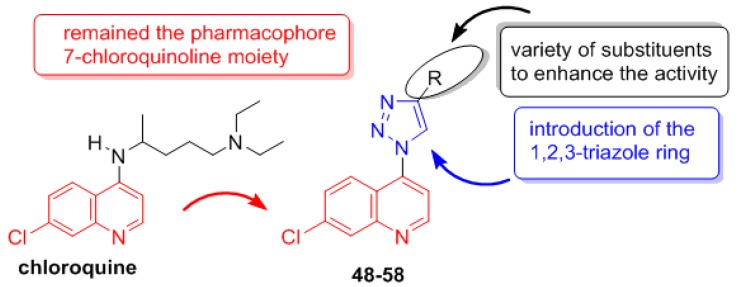
Rational approach to the design of quinoline derivatives **48**–**58**.

The literature describes several compounds containing a 1*H*-1,2,3-triazole ring with *P. falciparum* activity [37]. Previous works described by our group demonstrated that 1,2,3-triazoles are active against *Mycobacterium tuberculosis* [38] and *Leishmania amazonensis* [39]. Therefore, it was planned to connect this ring to the 7-chloroquinoline moiety present in CQ and amodiaquine and incorporate a variety of substituents at the 4 position (Figure 8).

**Figure 9:**
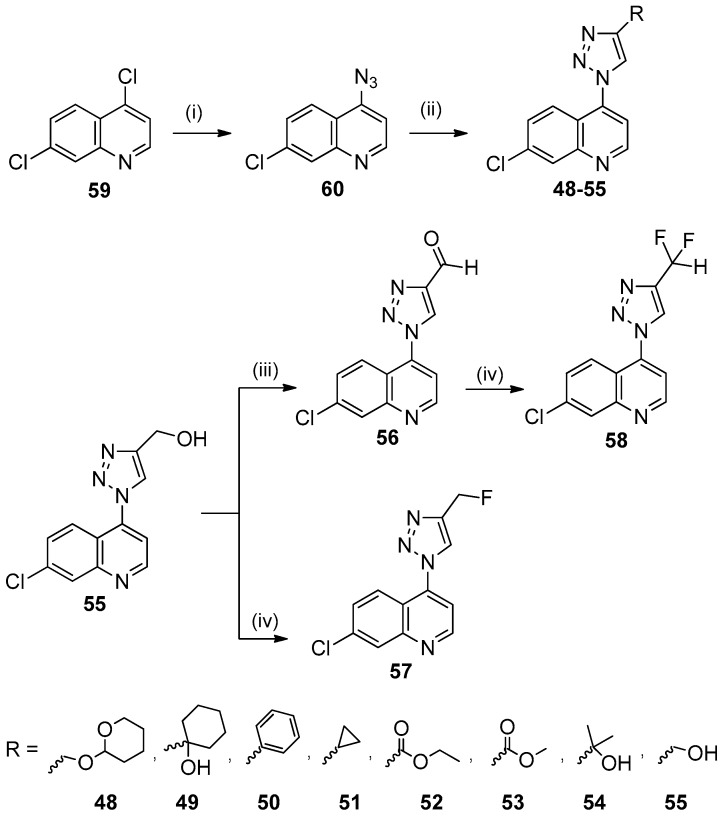
Synthesis of 7-chloro-4-(1*H*-1,2,3-triazol-1-yl)quinolines **48**–**58**. *Reagents and conditions*: (i) NaN_3_, MeOH, reflux, 24 h; (ii) appropriate acetylene, L-ascorbic acid sodium salt, CuSO_4_.5H_2_O, H_2_O/t-BuOH/THF (1:1:1), 25 °C, 24 h; (iii) ClCOCOCl, CH_2_Cl_2_, DMSO, TEA, −78 °C, 6 h; (iv) DAST, CH_2_Cl_2_, 25 °C, 24 h.

The synthetic route that was used to prepare the 7-chloro-4-(1*H*-1,2,3-triazol-1-yl)quinolines **48**–**58** is shown in Figure 9.

The raw material 4,7-dichloroquinoline (**59**) was treated with sodium azide in MeOH under reflux for 24 h to obtain the 4-azido-7-chloroquinoline (**60**) derivative in 70% yield. The 1,3-dipolar cycloaddition reaction between azide derivative **60** and the respective alkyne was performed with sodium ascorbate and a Cu(I) catalyst, in H_2_O/*t*-BuOH/THF (1:1:1) at 25 °C to obtain the 1,4-regioisomer of 7-chloro-4-(1*H*-1,2,3-triazol-1-yl)quinolines **48**–**58** in 40–77% yield [38].

Alcohol derivative **55** was used for the synthesis of compounds **56**, **57** and **58**. Aldehyde **56** was prepared in 55% yield, by the Swern oxidation of **55**. The reaction of **55** with dimethylaminosulfur trifluoride (DAST) in CH_2_Cl_2_ at 25 °C for 24 h produced the monofluorinated derivative **57** in 70% yield. The difluorinated derivative **58** was obtained with 60% yield from the reaction of aldehyde **56** with DAST.

We synthesized eleven new hybrid 7-chloro-4-(1*H*-1,2,3-triazol-1-yl)quinolines **48**–**58**. Six compounds, **48**, **50**–**52**, **56** and **58**, exhibited in vitro activity against the *P. falciparum* W2 CQR clone, with IC_50_ values ranging from 1.4 to 46 μM. None of these compounds were toxic to HepG2 cells.

**Figure 10:**
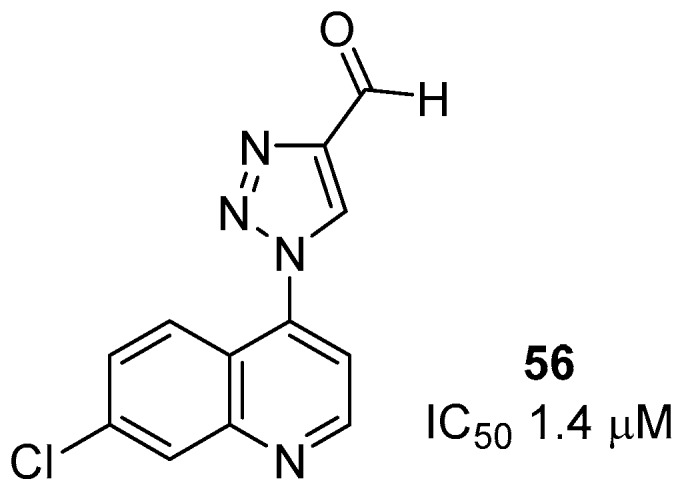
Structure of compound **56**.

The most active compound **56** (Figure 10) contained an aldehyde group at the 4 position of 1*H*-1,2,3-triazol-1-yl, with an IC_50_ = 1.4 μM and SI = 351.

Hybrid antimalarial drugs have advantages over combined drugs since they decrease the risk of adverse drug-drug interactions and facilitate treatment adherence. Through a rational drug design approach, single hybrid molecules with dual functionality and/or targets have been planned and may have either the same mechanism of action as the precursor drugs or a distinct mechanism of action [40,41].

**Figure 11:**
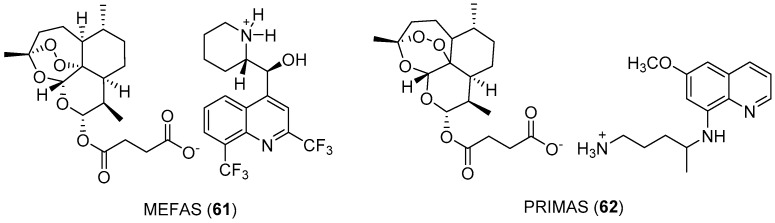
Structures of MEFAS and PRIMAS.

A hybrid salt of artesunate and mefloquine, MEFAS (**61**) (Figure 11), was active against both the CQS 3D7 and CQR W2 strains of *P. falciparum*, with IC_50_ = 0.001 μM. This hybrid salt was at least 5-fold more potent than mefloquine alone, more potent than artesunate against 3D7, as effective as artesunate against W2, and more potent than mixtures of the drugs. In vivo tests against *P. berghei*-infected mice, at a dose of 10 mg/kg, led to cure without recrudescence of parasitemia. The in vivo cytotoxicity of MEFAS has demonstrated that its toxicity is 5-fold lower than that of mefloquine. The combined fixed dose of ASMQ (artesunate + mefloquine) was 3-fold more toxic than MEFAS [42]. MEFAS has been demonstrated to be an active blood schizonticidal and can also block the infectivity of *P. falciparum* gametocytes, 280- and 15-fold more effectively than mefloquine and artesunate alone, respectively [43]. These results make this compound very promising to target both the asexual parasites and gametocytes, improving the antimalarial effects.

PRIMAS (**62**) (Figure 11) is also a hybrid salt between artesunate and primaquine under development by Boechat and coworkers [44]. This hybrid was designed with the goal of minimizing primaquine toxicity. Indeed, the PRIMAS hybrid salt is more active in vivo and in vitro and less toxic than primaquine.

Hybrid compounds that have been designed through the incorporation of different pharmacophoric groups into the quinoline ring have generated effective derivatives. Some are already in the clinical trial phase [45].

**Figure 12:**
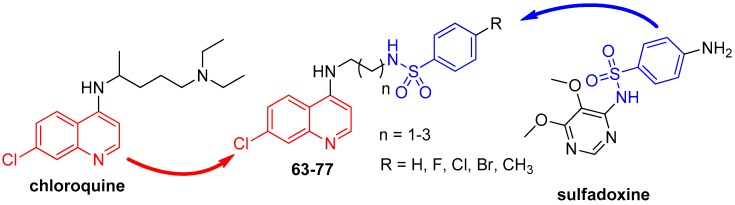
Rational approach to the design of compounds **63**–**77**.

In an effort to enhance the anti-*P. falciparum* activity of quinoline derivatives **63**–**77**, a series of 15 molecules was designed from the precursor drugs chloroquine and sulfadoxine (Figure 12) [46]. These compounds contain the pharmacophore groups of a 7-chloroquinoline and arylsulfonamide, separated by a distinct linker that is not found in the individual drug molecular frameworks. The 7-chloroquinoline moiety was included because it is present in CQ, which is used to treat malaria, while the arylsulfonamide moiety is present in sulfadoxine. The pharmacophore groups were connected by a linker group containing 2–4 CH_2_ units.

**Figure 13:**
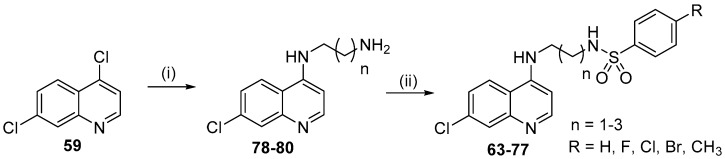
Synthetic route used to prepare compounds **63**–**77**. *Reagents and conditions*: (i) appropriate diamine, reflux, 4 h; (ii) appropriate sulfonyl chloride, MeOH, TEA, 25 °C, 24 h.

The synthetic route to prepare the *N*-(2-((7-chloroquinolin-4-yl)amino)alkyl)benzenesulfonamides **63**–**77** is shown in Figure 13.

The nucleophilic substitution reaction of 4,7-dichloroquinoline (**59**) with the corresponding diamine was performed to obtain intermediates *N*^1^-(7-chloroquinolin-4-yl)alkyldiamine **78**–**80** in 85-90% yield [47]. The addition-elimination reaction between the *N*^1^-(7-chloroquinolin-4-yl)alkyl-diamines **78**–**80** and the appropriate sulfonyl chloride in MeOH and TEA at 25 °C afforded the *N*-(2-((7-chloroquinolin-4-yl)amino)alkyl)benzenesulfonamides **63**–**77** in 50–77% yield [48].

The 15 synthesized compounds **63**–**77** with different substituents at the 4 position of the arylsulfonamide group were tested against the W2 chloroquine and sulfadoxine-resistant *P. falciparum* clone and for their cytotoxicity.

All the compounds presented activity, and 10 of them showed IC_50_ values ranging from 0.05 to 0.40 μM in the anti-HPR2 assay, which are lower than those of CQ and sulfadoxine, and none of them were toxic to BGM cells.

A direct relationship was observed between the increase in the number of methylene groups (CH_2_) used as the linker and the increase in activity. Compounds with 4 and 3 methylene group (CH_2_) linkers showed greater activity against *P. falciparum* than CQ, while the series of compounds containing 2 methylene groups were less active than CQ, with IC_50_ values in the range of 0.48 to 1.63 μM. Compounds **68**–**72**, with three methylene groups as the linker, showed IC_50_ values ranging from 0.10 to 0.35 µM. Compounds **74**–**77** containing four methylene groups as the linker were the most active of the series, with IC_50_ values ranging from 0.05–0.15 µΜ. Compound **73** (R = H) (IC_50_ = 0.40 µΜ) was an exception. Compounds with substituents at the 4 position of the arylsulfonamide group increased the *P. falciparum* activity, while the derivatives without a substituent on the arylsulfonamide group were the least potent. Compounds **72** (R = F; IC_50_ = 0.10 µΜ), **74** (R = CH_3_; IC_50_ = 0.05 µΜ), **75** (R = Cl; IC_50_ = 0.09 µΜ) and **77** (R = F; IC_50_ = 0.15 µΜ) had the highest SI values: 3386.0, 2489.0, 1102.2 and 1031.3, respectively, so they were also evaluated for their antimalarial activity in *P. berghei*-infected mice.

Compounds **74** and **77** were partially active and inhibited *P. berghei*-parasitemia by 27% and 30%, respectively on day 5 upon treatment at 10 mg/kg, administered orally, whereas compounds **72** and **75** showed parasitemia inhibition at rates of 47% and 49%, respectively.

**Figure 14:**
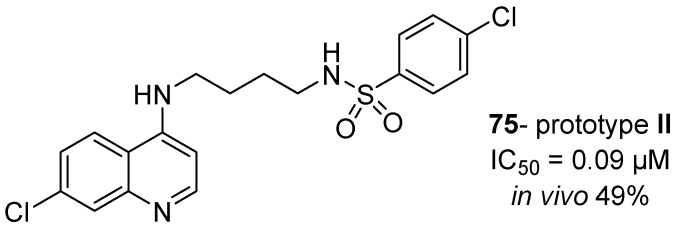
Structure of prototype **II**.

Due to the advantages of the hybrids with the best results exhibited herein, compound **75** (Figure 14) was used as an antimalarial prototype (prototype **II**) to proceed with other studies to overcome the burden of resistance in *P. falciparum*.

**Figure 15:**
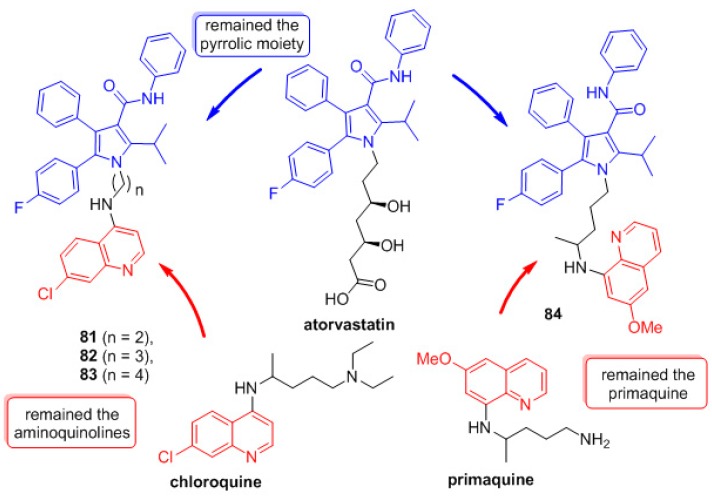
Rational approach to the design of compounds **81**–**84**.

Continuing the search for new quinoline derivatives with antimalarial activity, a new class of hybrids **81**–**84** with atorvastatin (AVA) was planned inspired by prototype II [49]. The design of these molecules was based on the proven AVA antimalarial activity. The molecular hybridization of chloroquine derivatives **81**–**84** included the aminoquinoline moiety with the pyrrole of AVA. To connect these two pharmacophoric groups, the 7-chloroquinoline moiety was bound to the pentasubstituted pyrrole by a linker group containing 2–4 CH_2_ units. Hybrid **84** was made by the direct attachment of primaquine to the pentasubstituted pyrrole (Figure 15).

**Figure 16:**
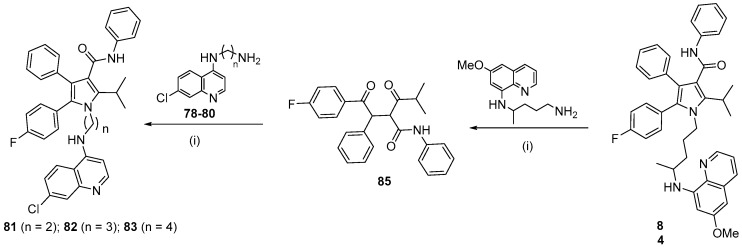
Synthetic route used to prepare compounds **81**–**84**. *Reagents and conditions*: (i) pivalic acid, THF: cyclohexane, 80–100 °C, 18–64%.

The Paal–Knorr [50] reaction between the key intermediate used in prototype II synthesis [47] *N*-(7-chloroquinolin-4-yl)alkyldiamines **78**–**80** or primaquine and 1,4-diketone **85**, new pyrrole-chloroquine **81**–**83** and pyrrole-primaquine **84** derivatives were obtained, respectively, in 18-64% yield (Figure 16). The *N*-(7-chloroquinolin-4-yl)alkyldiamines **78**–**80** were obtained as previously described [46].

**Figure 17:**
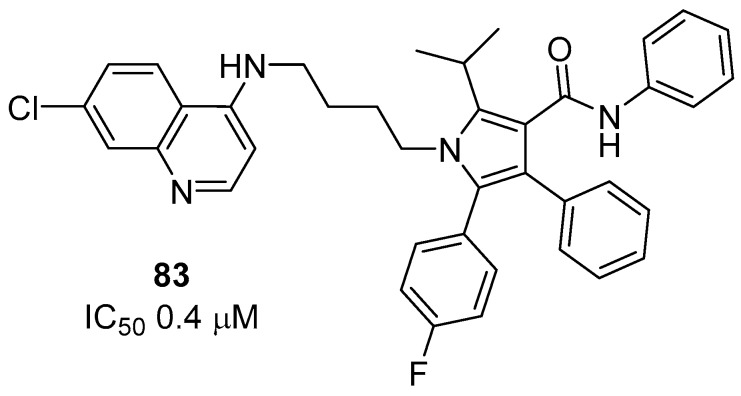
Structure of compound **83**.

All compounds synthesized showed activity against the *P. falciparum* W2 clone, and none of them were significantly toxic to the BGM cell line. Chloroquine-AVA hybrids **81**, **82** and **83** were the most active, with IC_50_ values of 0.99, 0.65 and 0.40 µM, respectively, which were in a similar range to chloroquine (IC_50_ = 0.59 µM), and all were better than primaquine (IC_50_ = 1.89 µM) and atorvastatin (IC_50_ = 10.3 µM), with good SI values. Herein, the activity of the compounds increased with the length of the carbon chain, which was similarly shown for the quinoline-sulfonamide derivatives that have four methylene groups as the linker. Compound **83** (*n* = 4) (Figure 17) exhibited better activity despite the exchange of the terminal amine for a polysubstituted pyrrole ring and was 26-fold more active than AVA. Primaquine derivative **84** (IC_50_ = 1.41) was the least potent of the series. However, it was significantly less toxic and more active than primaquine and 7-fold more active than AVA (IC_50_ = 10.3 µM). This indicates that compound **84** is promising and may be a prototype in the search for a drug that is able to replace primaquine, because **84** was shown to be safer (SI > 1107) than primaquine (SI of 239). Additionally, to evaluate the importance of the pyrrolic moiety in the hybrid compounds, a pentasubstituted pyrrole without the aminoquinolinyl moiety was synthesized. This compound had no toxicity in vitro and was more potent (IC_50_ = 6.39 µM) than AVA. This result suggests that the pentasubstituted pyrrole might be the pharmacophoric group of AVA for antimalarial activity. New compounds containing pentasubstituted pyrrole-quinolines will be synthesized with the expectation of enhanced potency and solubility and will also be assayed for cerebral antimalarial activity to clarify the importance of AVA in this scaffold.

**Figure 18:**
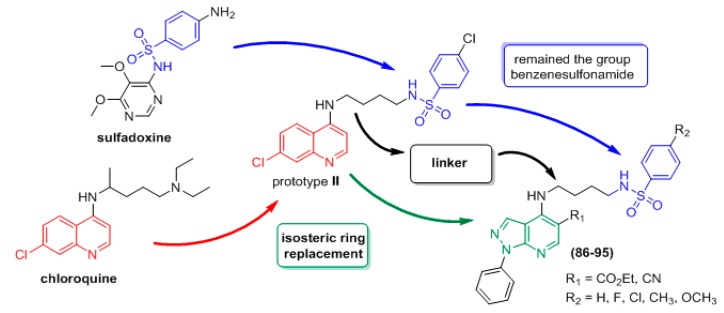
Rational approach to the design of compounds **86**–**95**.

In an effort to enhance the anti-*P. falciparum* activity of prototype **II**, new non-quinoline derivatives **86**–**95** were planned [51]. Therefore, the pyrazolopyridine system was selected as an isostere of quinoline, and the literature has demonstrated that this heterocycle possesses antimalarial activity [52]. The design of the non-quinoline derivatives consists of a ring isosterism in which the 7-chloroquinoline moiety is replaced by the 1-phenyl-1*H*-pyrazolo[3,4-*b*]pyridine system. An *N*-(4-aminobutyl)benzenesulfonamide group was attached to the 4 position of this heterocyclic ring. The 1*H*-pyrazolo[3,4-*b*]pyridine ring remains separated from the benzenesulfonamide moiety by the linker containing four methylene groups, which is similar to the linker found in the individual molecular framework of prototype **II** (Figure 18).

**Figure 19:**
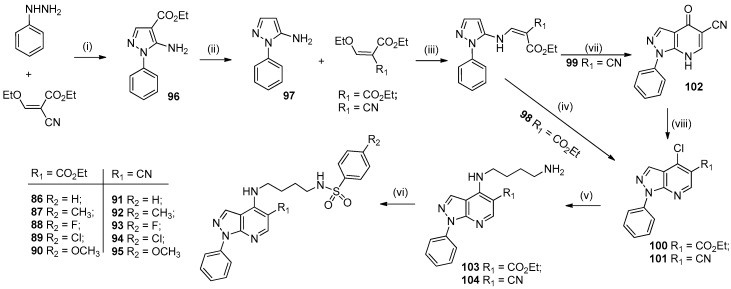
Synthetic route used to prepare compounds **86**–**95**. *Reagents and conditions*: (i) EtOH, reflux, 1 h, 70%; (ii) H_3_PO_4_, 170 °C, 6 h, 86%; (iii) EtOH, reflux, 2 h, 86–88%; (iv) POCl_3_, reflux, 6 h, 84%; (v) butane-1,4-diamine, 1,4-dioxane, 25–80 °C, 1–4 h, 66–79%; (vi) appropriate sulfonyl chloride, MeOH, TEA, 25 °C, 24 h, 59–70%; (vii) Dowtherm, 250 °C, 40 min, 71%; (viii) POCl_3_, reflux, 6 h, 90%.

The synthetic route for preparing the *N*-(4-((1-phenyl-1*H*-pyrazolo[3,4-*b*]pyridin-4-yl)amino)butyl)benzenesulfonamides **86**–**95** is shown in Figure 19.

From the reaction of phenylhydrazine and ethyl 2-cyano-3-ethoxyacrylate, in ethanol under reflux, the ethyl 5-amino-1-phenyl-1*H*-pyrazole-4-carboxylate (**96**) was prepared at 70% yield. Hydrolysis followed by decarboxylation of this compound afforded 5-amino-1-phenyl-1*H*-pyrazol (**97**) in 86% yield. Michael addition of **97** with ethyl 2-cyano-3-ethoxyacrylate or diethyl 2-(ethoxymethylene)malonate in ethanol under reflux gave derivatives **98** and **99** in 86% and 88% yield, respectively. From the reaction of diethyl 2-(((1-phenyl-1*H*-pyrazol-5-yl)amino)methylene)malonate (**98**) with phosphorus oxychloride under reflux, the intermediate ethyl 4-chloro-1-phenyl-1*H*-pyrazolo[3,4-*b*]pyridine-5-carboxylate (**100**) was prepared in 84% yield [53]. However, the derivative 4-chloro-1-phenyl-1*H*-pyrazolo[3,4-*b*]pyridine-5-carbonitrile (**101**) could not be obtained using this methodology. To obtain **101**, the cyclization of ethyl 2-cyano-3-((1-phenyl-1*H*-pyrazol-5-yl)amino)acrylate (**99**) was performed in refluxing Dowtherm for 40 min and 4-oxo-1-phenyl-4,7-dihydro-1*H*-pyrazolo[3,4-*b*]pyridine-5-carbonitrile (**102**) was isolated by precipitation from hexane with a yield of 84% [20]. Derivative **102** was treated with phosphorus oxychloride to produce **101** in 90% yield. From the nucleophilic substitution reaction between 4-chloro-1-phenyl-1*H*-pyrazolo[3,4-*b*]pyridines **100** and **101** and butane-1,4-diamine, the intermediates ethyl 4-((4-aminobutyl)amino)-1-phenyl-1*H*-pyrazolo[3,4-*b*]pyridine-5-carboxylate (**103**) and 4-((4-aminobutyl)amino)-1-phenyl-1*H*-pyrazolo[3,4-*b*]pyridine-5-carbonitrile (**104**) were synthesized in 79% and 66% yield, respectively [54]. The addition-elimination reaction between the appropriate sulfonyl chloride and amines **103** and **104** was performed in MeOH and TEA (1.0 mmol) at 25 °C to obtain target compounds *N*-(4-((1-phenyl-1*H*-pyrazolo[3,4-*b*]pyridin-4-yl)amino)butyl)benzenesulfonamides **86**–**95** in 59–70% yield [46].

Ten derivatives of 1-phenyl-1*H*-pyrazolo[3,4-*b*]pyridine **86**–**95** were synthesized with different substituents at the 4 position of the benzenesulfonamide group and tested in vitro against the W2 chloroquine and sulfadoxine-resistant *P. falciparum* clone and for their cytotoxicity. All the compounds exhibited low toxicity to BGM cells and IC_50_ values lower than that of the control drug sulfadoxine (IC_50_ = 15.0 µM). However, the observed activity was lower than that of chloroquine (IC_50_ = 0.55 µM) and the quinoline-sulfonamide hybrid **II**.

**Figure 20:**
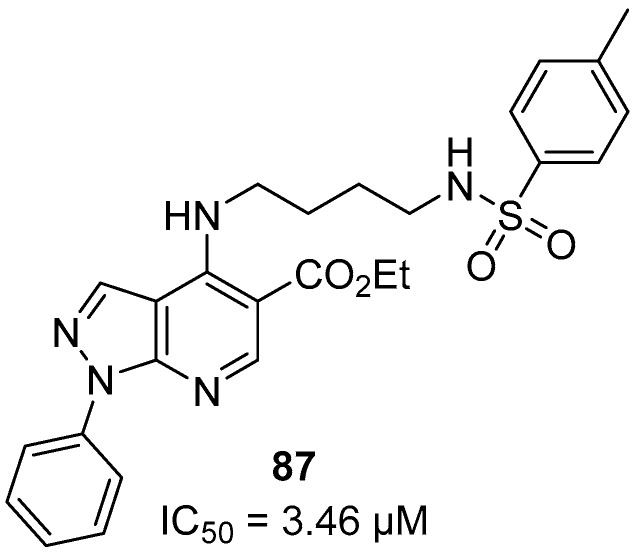
Structure of compound **87**.

The IC_50_ values ranged from 3.46 to 9.30 µM in the anti-HPR2 assay. Derivative (R_2_ = CH_3_) **87** with IC_50_ = 3.46 µM, derivative **89** (R_2_ = Cl) and **92** (R_2_ = CH_3_), both with IC_50_ values of 3.60 µM, were the most active against *P. falciparum* (Figure 20). Compound **89** also showed the highest SI value: >277.77.

It was observed that the 3 compounds with R_1_ = CO_2_Et at the 5 position of the 1*H*-pyrazolo[3,4-*b*]pyridine ring showed higher activity than those with CN at the same position.

**Figure 21:**
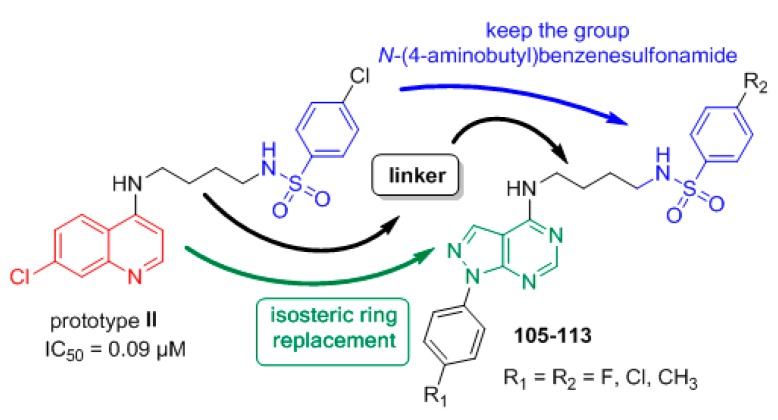
Rational approach to the design of compounds **105**–**113**.

Continuing the search to obtain new non-quinoline antimalarials, we used prototype **II** to design the new derivatives *N*-(4-((1-phenyl-1*H*-pyrazolo[3,4-*d*]pyrimidin-4-yl)amino)butyl)benzenesulfonamides **105**–**113** [55]. The 7-chloroquinoline moiety was replaced by the 1*H*-pyrazolo[3,4-*d*]pyrimidine system by ring isosterism, and at the 4 position of the heterocyclic ring, the *N*-(4-aminobutyl)benzenesulfonamide moiety was attached, designing the 1*H*-pyrazolo[3,4-*b*]pyridine derivatives (Figure 21).

**Figure 22:**
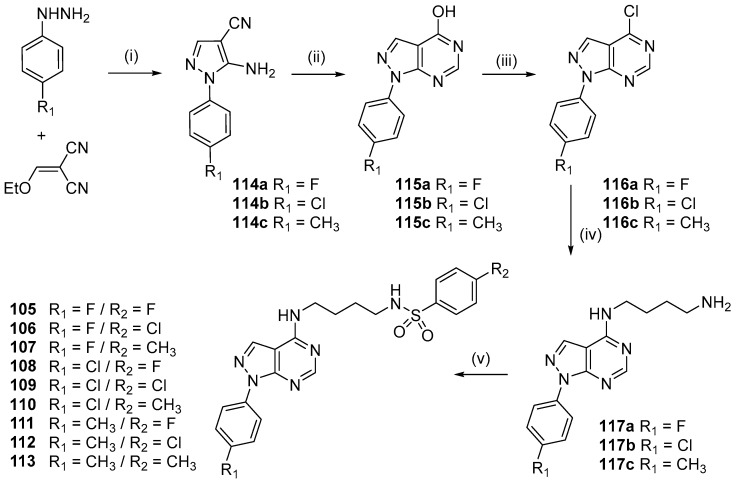
Synthetic route used to prepare compounds **105**–**113**. *Reagents and conditions*: (i) EtOH, reflux, 2 h, 61–80%; (ii) HCOOH, reflux, 12 h, 73–89%; (iii) POCl_3_, reflux, 24 h, 78–97%; (iv) butane-1,4-diamine, CH_3_CN, 25 °C, 24 h, 23–36%; (v) appropriate sulfonyl chloride, DMF, TEA, 90 °C, 24 h, 25–79%.

The synthetic route for preparing *N*-(4-((1-phenyl-1*H*-pyrazolo[3,4-*d*]pyrimidin-4-yl)amino)butyl)benzenesulfonamides **105**–**113** is shown in Figure 22.

**Figure 23:**
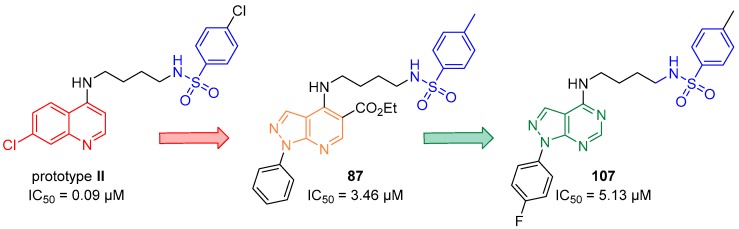
Comparison of prototype **II** with **87** and **107**.

The reaction of the appropriate phenylhydrazine and 2-(ethoxymethylene)malononitrile in ethanol under reflux for 2 h was performed to obtain the 5-amino-1-phenyl-1*H*-pyrazole-4-carbonitrile **114a**–**c** compounds in 61–80% yield [56]. Reaction of the suitable 5-aminepyrazoles **114a**–**c** and formic acid under reflux for 12 h afforded the 1-phenyl-1*H*-pyrazolo[3,4-*d*]pyrimidin-4-ols **(115a**–**c)** in 73–89% yield [57]. Derivatives **115a**–**c** were treated with phosphorus oxychloride under reflux for 24 h to produce 4-chloro-1-phenyl-1*H*-pyrazolo[3,4-*d*]pyrimidines **(116a**–**c)** in 78–97% yield [51,54]. Nucleophilic substitution between **116a**–**c** and butane-1,4-diamine in CH_3_CN at 25 °C for 24 h was performed to obtain intermediates *N*^1^-(1-phenyl-1*H*-pyrazolo[3,4-*d*]pyrimidin-4-yl)butane-1,4-diamines **(117a**–**c)** in 23–36% yield [46,51]. The addition-elimination reaction between diamines **117a**–**c** and the appropriate sulfonyl chloride in DMF and triethylamine (TEA) at 90 °C for 24 h afforded target compounds *N*-(4-((1-phenyl-1*H*-pyrazolo[3,4-*d*]pyrimidin-4-yl)amino)butyl)benzenesulfonamides (**105**–**113**) in 25–79% yield [46,51]. The nine derivatives of 1-phenyl-1*H*-pyrazolo[3,4-*d*]pyrimidine (**105**–**113**) that were synthesized were tested in vitro for their efficacy against the W2 chloroquine- and sulfadoxine-resistant *P. falciparum* clone and for their cytotoxicity. The anti-*P. falciparum* activities of the quinoline and 1*H*-pyrazolo[3,4-*d*]pyrimidine systems were then compared. Compounds **105**–**108**, **111**, and **113** showed IC_50_ values ranging from 5.13 to 43.40 µM in the anti-HPR2 assay and low toxicity to BGM cells. Among the compounds (**105–113**) synthesized, six compounds exhibited anti-*P. falciparum* activity in vitro against chloroquine-resistant parasites, and none were toxic to BGM cells. This study showed that compound **107** (R_1_ = F / R_2_ = CH_3_) presented an IC_50_ value of 5.13 µM, which was lower than that of the control drug sulfadoxine (IC_50_ = 15.00 µM) in the anti-HRPII assay. In addition, most of the compounds in this series have higher SI values than sulfadoxine. However, the 1*H*-pyrazolo[3,4-*d*]pyrimidine derivatives were not more potent than the control drug chloroquine (IC_50_ = 0.55 µM) and quinoline prototype **II** (IC_50_ = 0.09 µM). It is interesting to note that, similar to 1*H*-pyrazolo[3,4-*d*]pyridine **87**, the most active derivatives in this series have CH_3_ at the 4 position of the benzenesulfonamide moiety. Compounds 1*H*-pyrazolo[3,4-*d*]pyrimidine **107** and 1*H*-pyrazolo[3,4-*b*]pyridine **87** were equipotent; however, prototype **II** was still the most active (Figure 23). It is possible to conclude that the 1*H*-pyrazolo[3,4-*d*]pyrimidine and 1*H*-pyrazolo[3,4-*b*]pyridine systems are promising for further studies of their anti-*P. falciparum* activities.

**Figure 24:**
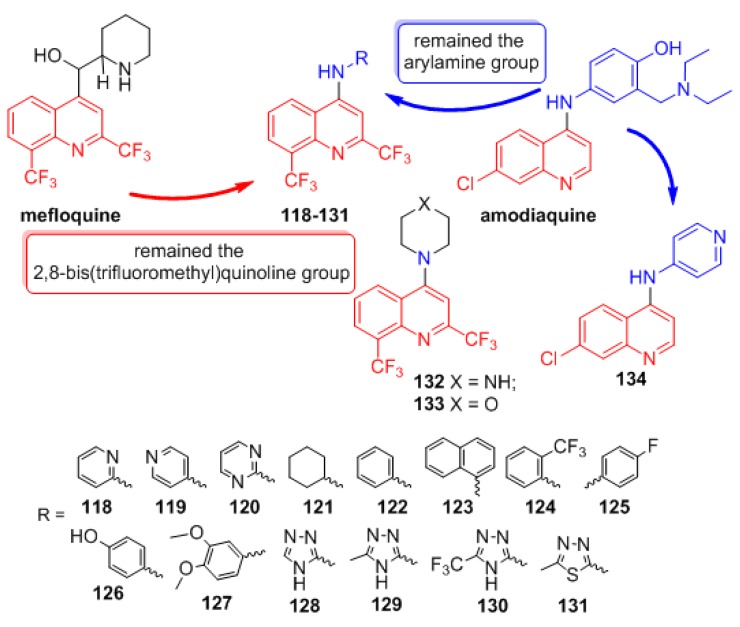
Rational approach to the design of compounds **118**–**134**.

New mefloquine derivatives **118**–**133** were designed using molecular hybridization and ring bioisosterism. The pharmacophoric subunit 2,8-bis-(trifluoromethyl)quinoline, which is present in mefloquine, and the aminoaryl moiety of amodiaquine were linked to provide potent antimalarial drugs (Figure 24) [58]. The importance of the arylmethanol moiety in mefloquine, also present in quinine, is highly important for the antimalarial activity however, the arylmethanol moiety was replaced by phenylamino group present in amodiaquine. A variety of aliphatic, aromatic and heteroaromatic substituents were added to provide the electronic and lipophilic properties to research the contributions of each fragment to the activity profile of this new class of compounds. Compound **134** was also prepared to compare the importance of the 2,8-bis-(trifluoromethyl)quinoline moiety versus the 7-chloroquinoline moiety for antiplasmodial activity.

**Figure 25:**
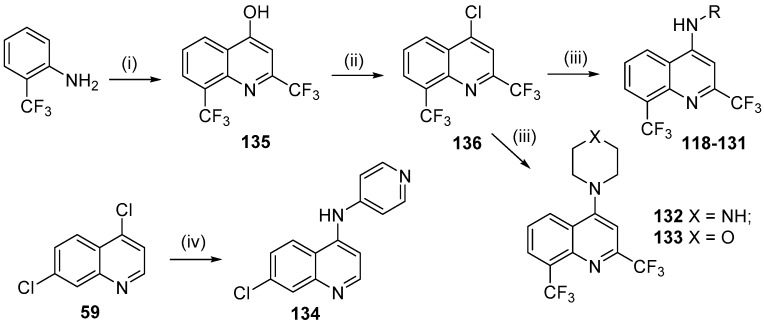
Synthetic route used to prepare compounds **118**–**134**. *Reagents and conditions*: (i) ethyl 4,4,4-trifluoroacetoacetate, PPA, 150 °C, 3 h; (ii) POCl_3_, 80 °C, 4 h; (iii) appropriate amine, NaH, DMSO, 25 °C, 1–24 h; (v) pyridin-4-amine, EtOH, 25 °C, 24 h.

The synthetic route to prepare the *N*-substituted-2,8-bis(trifluoromethyl)-quinolin-4-amine derivatives **118**–**133** is shown in Figure 25. Reaction of 2-(trifluoromethyl)aniline with ethyl 4,4,4-trifluoroacetoacetate in polyphosphoric acid (PPA) for 3 h produced 2,8-bis(trifluoromethyl)quinolin-4-ol (**135**) in 91% yield [59], which was then treated with phosphorus oxychloride at 80 °C to obtain 4-chloro-2,8-bis(trifluoromethyl)quinoline (**136**) in 98% yield [15].

The nucleophilic aromatic substitution reaction of intermediates **136** with the appropriate amine afforded target compounds **118**–**133** in 42–88% yield (Figure 24) [60].

Compound 7-chloro-*N*-(pyridin-4-yl)quinolin-4-amine (**134**) was obtained from the reaction of pyridin-4-amine with the commercially available 4,7-dichloroquinoline (**59**) in 89% yield [15].

**Figure 26:**
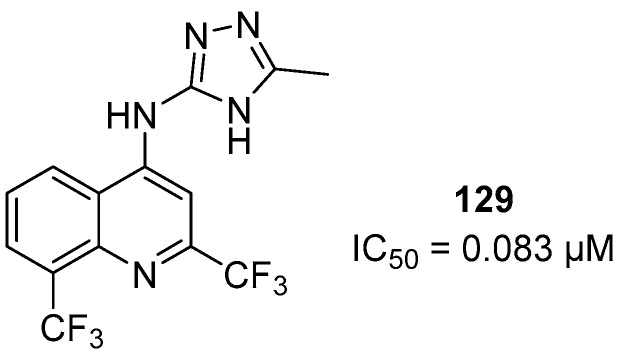
Structure of compound **129**.

First, a comparison was made between the importance of the 2,8-bis(trifluoromethyl)quinoline versus the 7-chloroquinoline moieties. Compounds **119** (IC_50_ = 8.4 ± 1.7 μM) and **134** (IC_50_ = 11.7 ± 3 μM) were equipotent, demonstrating that substitution of the quinoline core was not relevant for anti-*P. falciparum* activity. Compounds **132** (IC_50_ = 31.5 μM) and **133** (IC_50_ > 143 μM), which are both tertiary amines, were less active and inactive, respectively. This proves that the presence of the quinoline-NH group in this class of molecules is important. The reduction of anti-*P. falciparum* activity of **132** and **133** can be justified through the more rigid conformational structures of the cyclic tertiary amines compared to the arylamino derivatives. This rigidity interferes with the ability of the compounds to interact with the biomacromolecule receptor, although the NH group could still interact with the bioreceptor via hydrogen bonding. The most active compound **129** (IC_50_ = 0.083 µM) was 3-fold more potent than chloroquine (IC_50_ = 0.25 µM) (Figure 26). However, it was less active than mefloquine (IC_50_ = 0.019 µM). Moreover, as an advantage, its chemical structure is simpler than that of mefloquine because it does not contain a stereogenic center, and consequently, its synthesis in the laboratory is easier and less expensive. When the CH_3_ substituent was exchanged for H or CF_3_ on the triazole ring, as in **128** (IC_50_ = 2.9 µM) and **130** (IC_50_ = 11.5 µM), respectively, significantly lower activities were observed. The isosteric replacement of the 5-methyltriazole unit in **129** with the 5-methylthiadiazole unit in **131** (IC_50_ = 1.8 µM) decreased the activity, showing the importance of the triazole nucleus for anti-*P. falciparum* activity. Derivative **123** (IC_50_ = 9.6 µM), with the amine β-naphthyl group, was three times more active than **122** (IC_50_ = 28.0 µM), with the amine phenyl group, showing the importance of lipophilicity in this region on biological activity; however, **123** was not selective and showed high toxicity. Aminepyridin-4-yl derivative **119** (IC_50_ = 8.4 µM) was 2.5-fold more active and selective than aminepyridin-2-yl derivative **118** (IC_50_ = 19.6 µM). Moreover, when we evaluated the effect of the aminepyrimidin-2-yl derivative **120** (IC_50_ = 8.4 µM), it showed equipotent anti-*P. falciparum* activity and the same toxicity as **119**.

## 2. Conclusions

During our research on new drugs for malaria treatment, we sought out medicinal chemistry to make structural modifications and hybridizations to obtain new compounds that are more selective and less toxic. In the search of non-quinolinic compounds, a series of [1,2,4]triazolo[1,5-*a*]pyrimidine derivatives were designed. These compounds exhibited anti-*P. falciparum* activities, and none of the compounds were toxic to HepG2 cells. Compound **2,** with the CF_3_ group at the 2 position and β-naphthylamine at the 7 position, was the most active, and it has an important contribution to anti-*P. falciparum* activity with IC_50_ = 0.023 µM. Derivative **2** was used as a prototype compound (prototype **I**) for future investigations in the search for compounds for the treatment of malaria. Bioisosteric replacements of the triazolo[1,5-*a*]pyrimidine ring on prototype **I** by the pyrazolo[1,5-*a*]pyrimidine ring were shown to be effective. Derivatives with β-naphthylamine at the 7 position were the most active. Compounds **33** (R_1_ = CF_3_, R_2_ = CH_3_) and **38** (R_1_ = CH_3_, R_2_ = CH_3_) exhibited low toxicity and the low IC_50_ values of 1.2 and 5.1 µM, respectively, and consequently the highest SI values of 467.8 and 79.6, respectively. These compounds showed a 50% reduction of parasitemia in the in vivo anti-*P. berghei* malaria evaluation. Compound **44** (R_1_ = CH_3_, R_2_ = CF_3_) showed the best inhibition of the enzyme *Pf*DHODH with an IC_50_ = 0.16 µM, which was more potent than prototype **I** (IC_50_ = 0.70 µM). 

A hybrid salt of artesunate and mefloquine, MEFAS (**61**), has been demonstrated to be 280- and 15-fold more effective than both mefloquine and artesunate alone, respectively, against *P. falciparum* gametocytes. Against *P. berghei*-infected mice, a dose of 10 mg/kg led to cure, and the toxicity of **61** was 5-fold lower than that of mefloquine. PRIMAS (**62**) is also a hybrid salt between artesunate and primaquine. This hybrid salt is more active in vivo and in vitro and less toxic than primaquine.

The 1,2,3-triazol-1-yl quinoline derivatives exhibited activity in vitro against the *P. falciparum* W2 CQR clone without toxicity to HepG2 cells. The most active compound **56** showed an IC_50_ = 1.4 µM, and SI = 351. Compound **75** (R = Cl; IC_50_ = 0.09 µΜ) had an SI value of 1102.2 and was evaluated for its antimalarial activity in *P. berghei*-infected mice, showing 49% inhibition of parasitemia. This compound was used as an antimalarial prototype (prototype **II**) to proceed with other studies to overcome the burden of resistance in *P. falciparum*. From prototype **II**, derivatives 1*H*-pyrazolo[3,4-*d*]pyrimidine and 1*H*-pyrazolo[3,4-*b*]pyridine were designed. These cores were active against *P. falciparum* with **87** giving an IC_50_ = 3.46 µM and **107** showing an IC_50_ = 5.13 µM, which were lower than that of the control drug sulfadoxine; however, prototype **II** was still the most active. Chloroquine/primaquine-atorvastatin (AVA) hybrids showed similar activity to chloroquine but were better than primaquine. The primaquine derivative was significantly less toxic and more active than primaquine, 7-fold more active than AVA and was shown to be safer (SI > 1107) than primaquine (SI of 239). The 2,8-bis-(trifluoromethyl)quinoline derivatives are chemical structures simpler than those of mefloquine. Compound **129**, the most active compound, showed an IC_50_ = 0.083 µM and was 3-fold more potent than chloroquine (IC_50_ = 0.25 µM). We can conclude that these compounds are promising for further studies of antimalarial. However, the antimalarial activity of promising compounds to reverse artemisinin resistance can only be seen by establishing the ring-stage survival assay (RSA) [61].
